# Supplementary material for: A new metric for understanding hidden political influences from voting records
Source: PLoS One. 2020 Sep 1;15(9):e0238481. doi: 10.1371/journal.pone.0238481 (PMC7462714; doi:10.1371/journal.pone.0238481)
Supplement: Attachment — Submitted filename: reply_Rev1.pdf [file pone.0238481.s001.pdf]

Authors' reply to the Editor and Reviewers of the paper "A new metric for understanding hidden political influences from voting records"

Submission PONE-D-20-21006

July 30, 2020

Dear Editor,

We warmly thank you for your editorial assistance and we kindly ask you to consider our revised manuscript titled "A new metric for understanding hidden political influences from voting records" for publication in PLOS ONE as a research article.

This paper is a revised version of the first submission PONE-D-20-21006. In the previous round of revision, we received two high quality reviews and the overall recommendation was positive, with suggestions of some improvements and modifications by Reviewers. We carefully addressed the Reviewers' comments, and we feel that the paper largely benefited from the review process.

In the sequel we report the Reviewers' comments and suggestions followed by our replies and remarks, on a point to point basis.

We thank you in advance for the attention you will pay to our manuscript and we look forward to hearing from you. We certify that the current submission is original work and it is not under review at any other publication. All co-authors have seen and agree on the contents of the manuscript.

Best regards,

Corrado Possieri, Chiara Ravazzi, Fabrizio Dabbene, Giuseppe C. Calafiore

Editor

Comment: Two reviews were collected both very positive, although both reviewers suggest minor improvements or request minor clarifications. For this reason, I invite the authors to prepare a minor revision and to address such comments.

Answer: We would like to thank the Editor for the constructive reviewing process that helped us improve the quality of the manuscript and for the supportive and motivating comments about our work. We have carefully considered all the comments from the Reviewers, and their suggestions have been incorporated into the revised version of the paper, as detailed below.

Reviewer 1

Comment: The authors must be complimented for a very interesting and novel application of a classical (but probably still the more straightforward and meaningful) technique as PCA. The strategy adopted by the authors allows for a consistent analysis of both q and c space giving a clear picture of both senator profile and bill relevance together with an appreciation of the the 'internal homogeneity' of the different political parties.
Answer: We thank the Reviewer for his appreciation of our the paper, for the motivating and supportive comments, and for the useful suggestions that helped us preparing this revised version of our paper.

Comment: I have only one (very minor) request of clarification that I think could be useful to the readers to fully appreciate the proposed methodology. The authors affirm:

> [...] Therefore, noise filtering through decreasing the values of q and c reveals subtle structures of the political affinities among voters [...]

This statement is strictly dependent from the use of SPCA instead of 'plain' PCA in which happens exactly the opposite ('subtle structures' are confined in minor components and filtering them out only maintains the 'global trends' see for example:

- Roden, J. C., King, B. W., Trout, D., Mortazavi, A., Wold, B. J., & Hart, C. E. (2006). Mining gene expression data by interpreting principal components. BMC bioinformatics, 7(1), 194., Censi, F., Calcagnini, G., Bartolini, P., & Giuliani, A. (2010).
- A systems biology strategy on differential gene expression data discloses some biological features of atrial fibrillation. PLoS One, 5(10), e13668., Giuliani, A., Colosimo, A., Benigni, R., & Zbilut, J. P. (1998).
- On the constructive role of noise in spatial systems. Physics Letters A, 247(1-2), 47-52.).

In any case this is only a very minor remark and the manuscript can be even accepted with no modifications at all.
Answer: The point raised by the Reviewer is very interesting and therefore we added a detailed explanation following his suggestion. Namely, we added the suggested references and following paragraph to the discussion at page 13 of the revised version of the manuscript:

> "It is worth noticing that this filtering property is the result of the usage of SPCA for dimensionality reduction. In fact, subtle structures are usually confined in minor components [29]-[31], and filtering them out usually only maintains the global trends. On the other hand, SPCA allows one to focus just on the most relevant votes, hence undercovering hidden influences among individuals with different ideologies."

Reviewer 2

Comment: The manuscript is very interesting and sound, I have few minor comments for the authors.

Answer: We thank the Reviewer for his/her appreciation of the paper, for the supportive comment, and for the useful suggestions that helped us preparing this revised version.

Comment: Reading the introduction of the paper I wasn't 100% sure that the bills used to conduct the analysis were those in which the vote is not secret. Even if it would be impossible to perform the analysis using secret votes I think that the Introduction should clearly state that only "public" bills were used and that those bills are just a part of the total ensemble.

Answer: We revised the introduction so to clarify the class of votes that have been taken into account in performing the analysis of the Italian Senate during the XVII Legislature. Namely, we stressed the fact that our focus are voting data that are classified by OpenPolis as *key votes*, i.e., those votes that are publicly available (non secret) and considered as the most important, both for the relevance of the subject matter and for the political value. We also acquired the nominal membership of each senator to her/his political group, which will be used as side information. We further stressed that these votes constitute just a portion of the total ensemble of votes made by a senator, which also include, e.g., the ones made in secret ballots.

Comment: The explanation of the visualization proposed in Figure 3 (that according to the authors is part of the novelty of the paper) is very brief and somewhat unclear. I understand the coordinates of the party and I also understand the presence of spikes proportional to π in the direction of the party. I don't understand why some shapes are resulting from the Figure, are those the result of lines going between adjacent and non-zero β values? If this is the case, what is the interpretation of such shapes? Plus, how this plot is different with respect to a radar plot? This drawbacks also affect Figure 5.

Answer: In the revised version of the paper, we better acknowledged that the Political Affinity Map is essentially a spider chart (usually referred to also as radar plot or Kiviat diagram) in which the length of each "spike" is proportional to the influence of the yth group to the ith senator and adjacent "spikes" are connected via a segment. Depending on the ordering of the political groups, this type of plots allows one to undercover the shift of each senator toward political parties different from the nominal one. For instance, larger areas of the polytope indicate that the ideology of the senator is influenced by several political parties, whereas unitary spikes in the direction of his/her nominal affiliation indicate that his/her ideology is consistent with the one of the party. Further, the Political Affinity Map allows one to determine senators with similar ideologies, political clusters, and the presence of outliers; see, e.g., [28].

Comment: Finally, Figure 3 and Figure 4 are basically two visualizations for the same thing. Similarities, differences and potential use of both (separately and combined) could be discussed in more detail.

Answer: In order to better discuss the relation between Political Affinity Maps and Segmentation Plots, we added the following paragraph at page 13 of the revised version of our paper:

> "It is worth noting that the Political Affinity Map and the Segmentation Plot are just two different graphical representations of the Political DNA. These two representations allows to gather different information about the ideology of senators. For instance, as already pointed out above, the Political Affinity Map is very useful to identify clusters and to detect the presence of outliers. On the other hand, the Segmentation Plot is very useful to undercover the reciprocal influence among different parties and to identify the leading groups. Therefore, the combined use of these two representation allows one to have a complete picture of the relationships among different parties."
